# Supplementary material for: A set of multi-entry identification keys to African frugivorous flies (Diptera, Tephritidae)
Source: Zookeys. 2014 Jul 24;(428):97–108. doi: 10.3897/zookeys.428.7366 (PMC4143993; doi:10.3897/zookeys.428.7366)
Supplement: Supplementary material 6 — Key to Ceratitis [file zookeys-428-097-s006.zip › SF6_ZooKeys_key to Ceratitis/key/SF6_ZooKeys_key to Ceratitis/Media/Html/Ceratitis oraria.htm]

***Ceratitis*** *(**Ceratalaspis**)
**oraria*** **De Meyer & Copeland**

*Ceratitis (Ceratalaspis) oraria* De Meyer
& Copeland, 2005: 1284.

Body
length. 4.94 (4.30-5.50) mm; wing length. 4.67 (4.35-5.00) mm.

Female

Head.
Antenna yellow to yellow-orange. First flagellomere 3 times as long as pedicel.
Arista with short to medium long rays at most twice as long as basal width of
arista; ventral rays shorter than dorsal rays basally. Frons yellow to
yellow-orange; with short scattered black setulae. Face white, lower half and below
lower eye margin more yellow. Genal seta and setulae orange to red, well
developed.

Thorax.
Postpronotal lobe pale yellowish white; with black spot. Scutum, ground colour
yellow-orange, with distinct black markings including median presutural and sutural
spots; prescutellar markings yellowish white, merged; with silver
microtrichosity and yellow pilosity. Scapular setae yellow. Scutellum white to
yellow, basally with small black spots, apically with three separate black
spots, extending to basal third. Subscutellum yellow with three separate black
spots, sometimes black spots touching along dorsal margin. Anepisternum yellow,
upper margin white with black patch; one black anepisternal seta, setulae pale.

Legs.
Yellow; setation yellow to orange except last tarsal segments black. Fore femur
with ventral setae orange.

Wing.
Bands mainly yellowish with some brown patches. Anterior apical and discal
bands not separated; subapical band free; posterior apical band absent;
crossvein R-M at, or just proximal to, middle of discal medial cell.

Abdomen.
Yellow to orange; pilosity mixed yellow and black. Tergites 2 and 4 with pale
grey band occupying posterior third of tergite. Tergite 5 with longer black
setulae along posterior margin. Oviscape about as long as preabdomen. Aculeus ten times longer than wide, tip slightly bilobed and
lateral margin slightly sinuate.

Male

As
female except for the following characters. Arista with medium long rays,
longest dorsal rays about 3 times as long as basal width of arista. Face
completely white. Fore femur posteriorly with longer and somewhat denser
pilosity.

(Description
after De Meyer & Copeland, 2005)
